# Human Error Prediction Using Heart Rate Variability and Electroencephalography

**DOI:** 10.3390/s22239194

**Published:** 2022-11-26

**Authors:** Nahoko Takada, Tipporn Laohakangvalvit, Midori Sugaya

**Affiliations:** 1 Hitachi, Co., Ltd., Tokyo 319-1292, Japan; 2 Shibaura Institute of Technology, Tokyo 135-8548, Japan

**Keywords:** human error, heart rate variability (HRV), electroencephalograph (EEG), stroop task

## Abstract

As human’s simple tasks are being increasingly replaced by autonomous systems and robots, it is likely that the responsibility of handling more complex tasks will be more often placed on human workers. Thus, situations in which workplace tasks change before human workers become proficient at those tasks will arise more frequently due to rapid changes in business trends. Based on this background, the importance of preventing human error will become increasingly crucial. Existing studies on human error reveal how task errors are related to heart rate variability (HRV) indexes and electroencephalograph (EEG) indexes. However, in terms of preventing human error, analysis on their relationship with conditions before human error occurs (i.e., the human pre-error state) is still insufficient. This study aims at identifying biological indexes potentially useful for the detection of high-risk psychological states. As a result of correlation analysis between the number of errors in a Stroop task and the multiple HRV and EEG indexes obtained before and during the task, significant correlations were obtained with respect to several biological indexes. Specifically, we confirmed that conditions before the task are important for predicting the human error risk in high-cognitive-load tasks while conditions both before and during tasks are important in low-cognitive-load tasks.

## 1. Introduction

Industrial applications of machine learning and deep learning as well as the performance improvement of computational equipment have been accelerating to replace human tasks with autonomous systems and robots in recent years. It is estimated that the global market of robotic process automation will reach almost USD 2 billion in 2021 [1]. As simple tasks are replaced by autonomous systems, the responsibility of handling more complex tasks is placed more often on human workers [2,3,4]. In the meantime, the situation in which work content will change before human workers become proficient in that work will arise more frequently due to rapid changes in business trends [5]. Consequently, it is more likely that human error in serious situations will increase, which emphasizes the need to effectively prevent human error.

Previous studies on human errors have been targeted at an improvement in workplace environments or work procedures in terms of human engineering and ergonomics [6,7,8,9]. Those studies proposed optimal workplace environments or work procedures that are less likely to cause human error, as well as factors for predicting human error probability or assessing human reliability [10,11,12]. In addition, it is also necessary to observe the psychological states that cause human error for each individual [13].

Questionnaires are often used to monitor the psychological state of an individual, such as the evaluation of stress/fatigue levels and the risky driving behavior of occupational drivers [14]. However, some arguments on the effectiveness of questionnaires for assessing psychological states have been pointed out. Human judgment in questionnaire evaluation may be affected by cognitive and motivational biases of the respondent’s unique personal experience or group identity [15]. In addition, questionnaires cannot monitor unconscious changes in psychological or cognitive state in advance or in real time, which does not work to prevent of human error before it occurs. Thus, other approaches for more accurate, real-time, and personal monitoring of psychological states are necessary for the prevention of human error.

Research on sensor-based detection and the estimation of human psychological states, such as stress level [16] and emotion [17,18,19,20,21,22,23], has been increasing along with the developments of biosensors. However, these studies did not clarify the relationship between cognitive load and human error and thus could not predict human error probability. Some studies have focused on the modeling for predicting human error occurrence with biological signals (e.g., HRV and EEG) [24,25]. However, the data analysis results only covered a limited number of brain regions or did not assess psychological states “before” the human error (i.e., the human pre-error state) occurrence, so the results could not be utilized to prevent human error.

Even though there are many studies related to the estimation of human psychological states and human error occurrence, analysis of the relationship between the human pre-error state and human error probability, which would be useful for the prevention of human error, is still insufficient. Some situations absolutely require the detection and prevention of human error, e.g., in nuclear power plants that can cause tremendous damages on a large scale if appropriate measures of human error are not performed.

Thus, the goal of this study is to clarify the characteristics of biological indexes that are useful for estimating psychological states, both before and during tasks, that are likely to cause human error. In order to achieve this goal, we analyzed biological (i.e., HRV and multi-channel EEG) indexes acquired before and during the Stroop task. As a result, we obtained significant correlations between several biological indexes (e.g., HRV indexes and EEG indexes from frontal, occipital, and temporal lobes) and the number of errors. We also clarified a relationship between the cognitive load of the tasks and different brain regions. Our experimental results confirmed that the before-task condition (the human pre-error state) is important for predicting the human error risk of high-cognitive-load tasks, while both before- and during-task conditions are important for low-cognitive-load task. The details of our experiment and analysis are presented in this paper.

## 2. Previous Studies on Human Errors from Affective Computing

Studies on the estimation of human psychological states using biological information are of great interest in the field of Affective Computing. There are two main approaches, which are discussed in the following paragraphs.

The first approach is the estimation of human psychological states that affect human error, such as fatigue, stress and cognitive load. From the viewpoint of human error prevention, Zhang et al. employed HRV and EEG indexes to evaluate the influence of fatigue on response time and error rate and suggested significant correlations between the response time after fatigue and the decrease in heart rate and increase in LF/HF [20]. Rodrigues et al. suggested that HRV indexes change significantly according to stress level [26]. Moreover, several studies have reported the correlations between cognitive load and HRV, EEG, and fNIRS indexes. John et al. measured several biological information (e.g., HRV, EEG, fNIRS, and GSR) during a simulator-based enemy airplane monitoring task and suggested significant correlations between cognitive load and fNIR and EEG indexes, employing a total of 20 biological indexes [21]. Shriram et al. suggested the possibility of using EEG indexes to estimate workload [27]. Durantin et al. employed fNIRS and HRV indexes to estimate the difficulty of mental workload task [28]. Muthukrishnan et al. suggested estimating cognitive capacity with HRV indexes [29]. Alba et al. suggested correlations between HRV and EEG indexes and cognitive flexibility [30]. Cha et al. also used EEG indexes to estimate teamwork by analyzing the multi-workers synchronization of EEGs [31]. Recent studies also employed EEG for various applications, especially for emotion recognition, and employed advanced techniques such as deep learning, for the modeling, which effectively captured the psychological states of human [22,23]. These studies revealed the correlations between biological indexes and human psychological states that may cause human error. However, the relationship with human error itself has not yet been discussed.

The second approach is the estimation of human error occurrence and the probability of such occurrence. This approach can be classified into two types. The first one focuses on the detection of human error occurrence. For this purpose, previous studies have employed EEG indexes to detect the human perceptions of human errors in the flanker task, the error awareness dot task (EADT), or the Stroop task [32,33,34,35]. The second focuses on the detection of error-prone states before or at the time of human error occurrence. Lin et al. proposed a classification model for predicting human error occurrence using EEG indexes collected from six electrodes (FC3, FCZ, C3, CZ, CP3, and CPZ) to monitor the motor and sensory cortexes used to control right-hand movements, which is related to the prediction of human errors in numerical typing [24]. They successfully classified human error by AUC 0.62 and detected human errors 300 ms before the error in key stroke occurred. However, the model cannot be generalized because it used EEG signals obtained from only a limited number of brain regions. Kishimoto et al. surveyed the influence of discomfort with workplaces on human errors using NASA-TLX [36] as a criterion for workplace environments to suggest a specific correspondence between the NASA-TLX and HRV indexes as the human error occurred and clarified the characteristics of HRV indexes at the moment of human error occurrence [25]. However, they cannot be utilized for preventing human error, which also requires the assessment of psychological states “before” the human error (i.e., the human pre-error state) occurrence. Smit et al. suggested correlations between EEG indexes at resting states and timing errors in finger tapping [37]. Kim et al. suggested HRV and EEG indexes, particularly lnLF and lnHF, for detecting human errors [38]. Oh et al. also employed HRV and EEG indexes to predict the probability of human error occurrence, but they reported that there were no significant differences between those biological indexes and error-prone and non-error states [39].

Table 1 summarizes the above-mentioned previous studies on using biological information for the estimation of human psychological states, human error probability, or human error occurrence. The attempt by Lin et al. [24] was closest to our goal, as they also considered the human pre-error state, but their analysis was based on only motor and sensory cortexes, while other brain regions related to cognitive process were not considered. This is insufficient for the estimation of human error, as it involves various factors including those not related to motor and sensory cortexes [40].

## 3. Experimental Method

### 3.1. Purpose

The purpose of the experiment was to analyze the correlation between the number of errors and the biological indexes acquired from HRV and EEG before and during cognitive tasks to clarify useful biological indexes for predicting human error probability.

### 3.2. Measuring Instruments

To acquire biological information, we used two types of biological sensors: (1) ECG sensors and (2) EEG sensors. The details are as follows:

For the ECG sensor, we employed myBeat (Union Tool Co., Tokyo, Japan), a multimodal sensor that can acquire electrocardiograph (ECG) signals with a 1000-Hz sampling rate, three-axis acceleration, and body surface temperature. The electrode was attached to the upper left chest to monitor the heart’s electrical activity. The pNNx indexes were obtained through the analysis of an R-R interval (RRI) in 30 heart beats, while the LF/HF was obtained from analytical software provided by Union Tool Co. [41].

**Figure 1:**
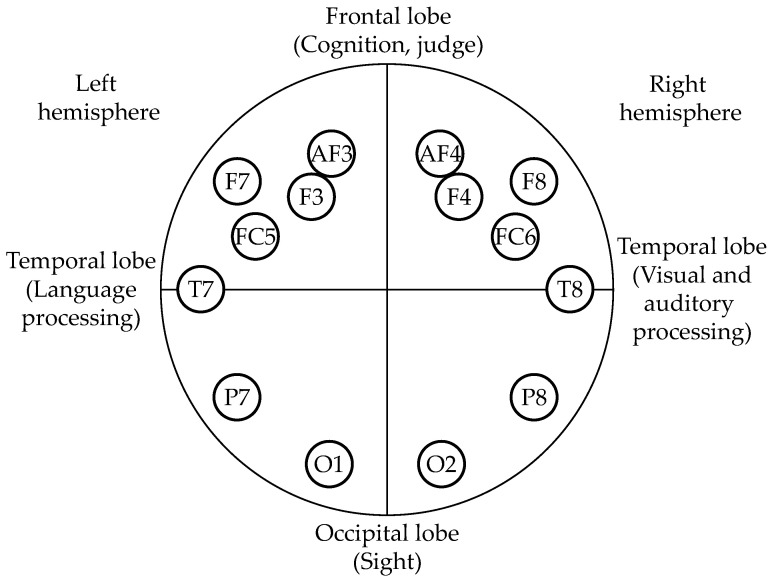
Position of the measuring electrodes [60] and assumed functional localization of the brain [53,54,55,56,57,58].

For the EEG sensor, we employed Epoc+ (Emotiv Inc., San Francisco, CA, USA), which is widely used as a low-cost research-purpose EEG sensor [42], providing 14 EEG channels (i.e., AF3, AF4, F3, F4, F7, F8, FC5, FC6, T7, T8, P7, P8, O1, and O2) by 10-10 systems (Figure 1) with a sampling frequency of 256 Hz. Using EmotivPRO, a dedicated software for Emotiv Epoc+, we could determine the signal quality of each EEG electrode as a score from 0 (bad) to 4 (good) based on the combination of these metrics calculated by Emotiv’s original algorithms: contact quality (CQ), machine-learning based signal quality (SQ), and signal magnitude quality (SMQ) [43]. Prior to the start of EEG recording, we confirmed that all 14 EEG electrodes were determined as “good”.

### 3.3. Processing of Biological Information

HRV and EEG indexes as described in the following sections were chosen from previous studies by Lin et al. [24] and Kishimoto et al. [25], as they investigated the estimation of psychological states related to human error. Since the combination of HRV indexes and EEG indexes have been successfully used for emotion estimation [18,19,44], we proposed using them in combination in order to estimate psychological states related to human error more accurately.

- HRV indexes

RRI and HRV obtained from the RRI were employed as HRV indexes. Previous studies suggested that the HRV indexes are related to parasympathetic nerve system (PNS) activity and sympathetic nerve system (SNS) activity [45,46,47]. HRV indexes are usually analyzed using two methods: time-domain analysis (e.g., pNNx) and frequency-domain analysis (e.g., LF/HF). The pNNx is the ratio of the heart rate whose differential in the neighboring RRIs is larger than x ms. A larger pNNx represents a state in which the parasympathetic nerve is more dominant [46,48]. pNN50 is one of the most widely used HRV indexes. In addition, some studies suggest that a pNNx whose x is not 50 shows a different sensitivity to the PNS activities [49]. LF/HF is the ratio of the low-frequency component (LF) to the high-frequency component (HF) of HRV, which corresponds to the balance of PNS and SNS activities [47,50]. The PNS and SNS activities reflect positive/relaxed emotions and negative/stressed emotions, respectively [18,19,51,52]. Thus, we employed a total of 14 HRV indexes consisting of the RRI, pNNx (where x = 10, 20,…, 100), LF, HF, and LF/HF for estimating human psychological states related to human error (Table 2).

- EEG indexes

Frontal, occipital, and temporal lobes of the brain regions were chosen as the target of EEG measurement. The frontal lobe plays an important role in recognition and judgement by activating other parts of the brains to perform an appropriate output to the response task [53]. The occipital lobe is related to visual processing [54]. The left temporal lobe is reported to accommodate the language center such as Wernicke’s area and Broca’s area [55], while the right temporal lobe is related to visual and auditory processing [56,57,58]. We employed five EEG signals in different frequency ranges consisting of Theta, Alpha, Low Beta, High Beta, and Gamma for estimating human psychological states such as drowsiness, concentration, arousal, and impatience (Table 3). Thus, we employed a total of 70 EEG indexes (5 EEG indexes, each of which has 14 channels). Data preprocessing (noise reduction and filtering) of the EEG was performed using EmotivPro software (Emotiv Inc., San Francisco, CA, USA).

**Table 3:** EEG indexes used in this study and their related frequencies and brain states [59].

EEG Index	Frequency (Hz)	Related Brain State
Theta	4–8	Relaxed, meditative, creative, memory recall and ‘flow’
Alpha	8–12	Relaxed and alert
Low Beta	12–18	Active, task-oriented, anxious thinking, and concentration
High Beta	18–25	
Gamma	>25	Demanding cognitive or motor functions

### 3.4. Cognitive Load Task

**Figure 2:**
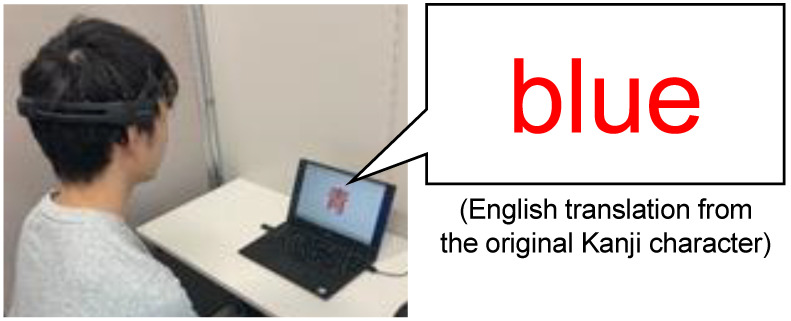
Experiment scene where a Kanji means “blue” is indicated on the screen in red ink.

The Stroop task [61] was employed for the observation of human errors induced by cognitive load. In the Stroop task, incongruent stimuli (e.g., a letter of “blue” printed in red ink, cf. Figure 2) is presented to generate high cognitive load, the so-called Stroop effect. It is a widely used method for the observation of human errors induced by working-memory capacity [62]. During response selection in the Stroop task, the human brain is involved in several complex processes all at once, such as processing speed, selective attention, automaticity, and parallel distributed processing [63]. Since we intended to use the Stroop task to induce high cognitive load, we set the response time for each question in the Stroop task to 1 s. This fast stimulus presentation while the brain is performing several complex processes is likely to induce a human psychological state conductive to high risk of human error, which is fundamentally important for further study with more complex tasks.

In the Stroop task, two pieces of information (word and ink color) are interfering. It is reported that the color answering task requires more time than the word reading task [64]. Dehaene et al. reported that the color answering task required high cognitive load and the activation of multiple brain regions [65]. Therefore, in the word reading task, the error is observed when the Stroop effect is low, meaning that cognitive load is low, whereas in the color answering task, the error is observed when the Stroop effect is high, meaning that cognitive load is high.

### 3.5. Experimental System Constructed for Human Error Estimation

To prevent human error, it is important to clarify the relationship between human error and biological indexes before the human error occurrence (pre-task condition). Thus, resting time before the Stroop task was set, and the biological information before human error was acquired.

**Figure 3:**
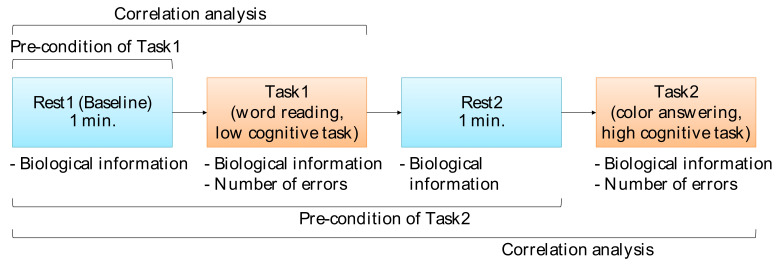
Order of rest and task phases and their relations for correlation analysis.

The Stroop task was then performed twice (Task1 and Task2) in the order shown in Figure 3. We regarded the difference in the number of errors between the two tasks as the strength of the Stroop effect that affected the participants. For all participants, we performed the experiment with the same task order (no counterbalanced), starting from Task1 (low-cognitive-load task) followed by Task2 (high-cognitive-load task) in order to control the task effect on the cognitive load. In addition, a resting period (Rest2) between Task1 and Task2 was also administered to prevent or reduce the participant’s tiredness after finishing Task1.

Correlation analysis between pre-task biological information and the number of errors, as well as correlation analysis between in-task biological information and the number of errors, were performed to clarify biological indexes related to human error probability. We regarded Rest1 as the pre-task phase of Task1, and Rest1, Task1, and Rest2 as the pre-task phases of Task2.

### 3.6. Experiment Procedure

One hundred tasks with a 1-s interval were set in each task phase, and the number of errors was measured. An error was defined as either an incorrect answer or no answer. To prevent noises from body and eye movements for biological information, the participants were asked to answer the task verbally. During the experiment, the number of errors was recorded, while the timing of error occurrence was not recorded. The period of each rest phase was 1 min. The experiment was performed in a silent room (Figure 2). Note that the room is not a shielded room designed specifically for the EEG measurement, so there may have been power noise that interfered with the recorded EEG signal.

The volunteered participants were 13 healthy men and 2 healthy women in their 20 s. As all of them are either native Japanese or non-native Japanese speakers residing in Japan, the experiment was conducted in Japanese including the content of the Stroop task in which a Japanese character (i.e., Kanji) was used. It has been reported that the words written in Kanji (logographic orthography) produced a greater Stroop effect than those written in Kana (phonetic orthography), as measured by fMRI [66]. Thus, we also employed Kanji in our Stroop task. (Note that there is no validated Japanese version of the Stroop test.)

The procedure of the experiment is as follows:

1. obtain consent for experiment cooperation;
2. attach ECG and EEG sensors;
3. explain the content of the experiment;
4. perform Rest1 for 1 min;
5. perform Task1 (reading words for 100 questions);
6. perform Rest2 for 1 min;
7. perform Task2 (answering colors for 100 questions).

### 3.7. Analysis Method

**Figure 4:**
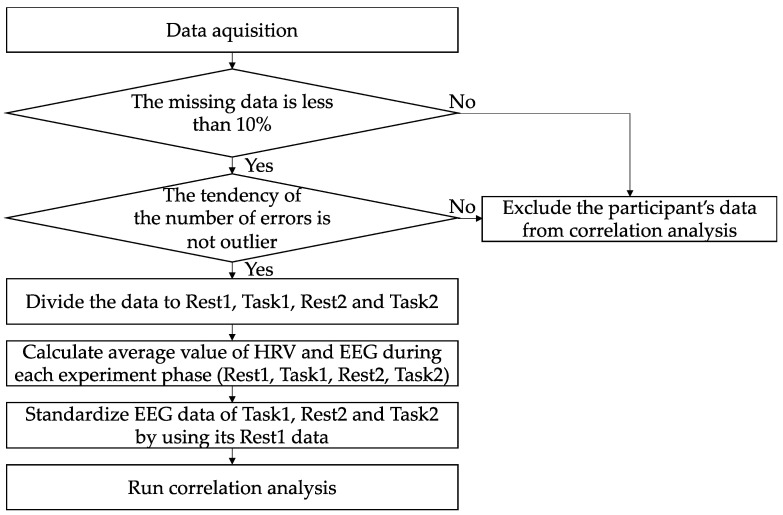
Flowchart of the data analysis method.

Figure 4 shows the flowchart of the data analysis method. We excluded the data of participants where more than 10% of the total data was missing or data with an abnormal tendency in the number of errors. As a result, the data of only 12 participants was used for the analysis. Despite of the small number of participants, we aim to analyze the data to obtain some clues on the order of importance among the biological indexes being employed in this preliminary experiment.

The data analysis was performed in the following 4 steps as follows:

(1) visualization of the number of errors per participant (Section 4.2);
(2) correlation analysis between the number of errors in Task1 and the biological indexes in Rest1 and Task1 (Section 4.3);
(3) correlation analysis between the number of errors in Task2 and the biological indexes in Rest1, Task1, Rest2 and Task2 (Section 4.4);
(4) correlation analysis between the difference in the number of errors between Task1 and Task2 and the biological indexes in Rest1, Task1, Rest2, and Task2 (Section 4.5).

Note that we only perform the analysis with the HRV indexes, but not with the EEG indexes during Rest1 because we used the EEG indexes during that period for data normalization.

Before selecting the correlation analysis method, we firstly checked if the data (i.e., the number of errors and the biological indexes) was normally distributed or not. Since we found that the data was not normally distributed, we employed a non-parametric test: Spearman’s rank correlation. In addition, since we aim to analyze the psychological states of each of the experiment conditions (Rest1, Task1, Rest2 or Task2) separately, we treated them as mutually exclusive instead of performing multiple comparisons.

The interpretation of correlation strength was regarded as shown in Table 4 [67]. As described in Section 3.3, 14 HRV indexes (RRI, pNN10–pNN100, LF, HF, and LF/HF) and the 70 EEG indexes (Theta, Alpha, Low Beta, High Beta, and Gamma in each of the 14 channels) were employed. The mean values of the biological indexes were used as criteria of the human psychological states. We used the mean values of EEG indexes during Rest1 as a baseline for the normalization of EEG data.

### 3.8. Human Use Protocols

The study has been reviewed and approved by the Ethics Committee of Shibaura Institute of Technology. The participants were recruited voluntarily. All of them were students of the Shibaura Institute of Technology. Written informed consent was obtained from the participants before performing the experiment.

## 4. Experimental Results

### 4.1. Overview

Our research goal was to clarify the characteristics of general-purpose biological indexes that can determine human error-prone states. To achieve this goal, we analyzed the correlation between the number of errors in the Stroop task and the mean values of the HRV and EEG indexes before and during the tasks.

For the analysis, we employed the data collected from 12 out of 15 participants consisting of 11 males and 1 female. The data from three participants were excluded for two reasons: (1) two of them had a problem with the measurement of biological information, causing about a 40% loss of data, and (2) one of them showed an opposite tendency in the error-prone state from that of all other participants (see Section 4.2); specifically, the number of errors in Task1 (low-cognitive-load word reading task) was much higher than that of Task2 (high-cognitive-load color answering task), which may have been caused by his/her being a non-native Japanese speaker. Therefore, we judged that the data from these participants were not suitable for further analysis.

### 4.2. Result 1: Number of Errors Generated by Each Participant

The distribution of the number of errors in Task1, Task2, and their difference (Task2–Task1) are shown in Table 5, Table 6 and Table 7, respectively. Task1 (word reading) shows an average number of errors of 1.8. Task2 (color answering) shows an average number of errors of 7.3. There were only three participants (B, D, and I), which is 25% of the participants, whose number of errors in Task2 (color answering) was the same as that of Task1 (word reading). As described in Section 3.4, since the Stroop effect of the word reading task is low, it can be concluded that the participants who had a higher number of errors in Task2 (C, F, G, J, K, L, M, N, and O) were affected by the Stroop effect.

The above results show that we were able to collect the data of two types of participants: those who were and those who were not sensitive to the Stroop effect. Long et al. also reported the different Stroop task performances due to the individual differences in working-memory capacity observable from incongruent tasks [68], which is similar to the high-cognitive-load color answering task (Task2) in our study.

### 4.3. Result 2: Correlation Analysis between the Number of Errors in Task1 (Low Cognitive Word Reading Task) and the Biological Indexes during Rest1 and Task1

We performed the correlation analysis between the number of errors in Task1 and the biological indexes during Rest1 (Table 8) and those of Task1 (Table 9). As described in Section 3.7, the correlation with EEG indexes during Rest1 was not analyzed because they were used for the normalization of EEG data during other experiment phases. For brevity, the tables only show the significant correlations.

As a result, we obtained moderate to strong correlations. As shown in Table 8 (the number of errors in Task1 vs. the biological indexes during Rest1), there were moderate positive correlations for pNN20, pNN30, pNN40, pNN50, pNN70, pNN80, pNN90, and HF and strong positive correlations for pNN10. As shown in Table 9 (the number of errors of Task1 vs. the biological indexes during Task1), there were moderate positive correlations for pNN80, pNN90, pNN100, and HF, strong positive correlation for LF, and moderate negative correlations for AF3 Alpha, AF3 Low Beta, P8 Theta, P8 Alpha, AF4 Theta, and AF4 Alpha. 

The above results indicate the tendency of an increased error during Task1 when the participants are relaxed before and during the task observed from positive correlations of pNNx and HF, and a decreased concentration in the task observed from the negative correlations of EEG indexes [59]. As described in Table 3, negative correlations from Theta can be regarded as less-flow, which means a less-concentrated condition. AF3 and AF4 are located at the frontal cortex which is related to cognition, and P8 which is related to the sensory function of visual, auditory, and tactile stimuli [53,58]. As for the positive correlation for LF during the task, there is a possibility that the LF was influenced by error recognition, since LF can be induced by positive and negative emotions [47]. 

To verify this possibility, we further analyzed HRV indexes: we divided Task1 into halves to analyze their correlations with the number of Task1 errors. The results are shown in Table 10 and Table 11 for the first half and the latter half, respectively.

There were moderate positive correlations for pNN80 and HF only in the first half of Task1. Since pNN80 and HF showed significant correlations during Rest1, the relaxed state was considered a factor contributing to the increase in the number of errors in Task1. On the other hand, there were moderate positive correlations for RRI and pNN10 only in the latter half of Task1, which shows the behavior that differs from the degree of relaxation. Since a larger RRI means a decrease in heart rate and pNN10 represents a lower variability than pNN80, these results may indicate the strength of sympathetic activity and the decrease in respiration due to the stress or tension caused by the error recognition during the task.

The above results show the possibility of estimating the state in which error is likely to occur in Task1 (low-cognitive-load word reading task) from the biological indexes as follows: the degree of relaxation using pNN10, pNN20, pNN30, pNN40, pNN50, pNN70, pNN80, pNN90, and HF before the task, the degree of relaxation using pNN80 and HF during the first half of task, and the degree of low concentration using AF3 Alpha, AF3 Low Beta, P8 Theta, P8 Alpha, AF4 Theta and AF4 Alpha during Task1.

### 4.4. Result 3: Correlation Analysis between the Number of Errors in Task2 (High Cognitive Color Answering Task) and Biological Indexes during Rest1, Task1, Rest2 and Task2

We performed a correlation analysis between the number of errors in Task2 and the biological indexes during Rest1, those of Task1 (Table 12), those of Rest2 (Table 13), and those of Task2 (Table 14). As described in Section 3.7, the correlations with EEG indexes during Rest1 were not analyzed because they were used for the normalization of EEG data during other experiment phases. There was no significant correlation with any HRV indexes during Rest1, Task1, or Task2.

As shown in Table 12 (the number of errors in Task2 vs. the biological indexes during Task1), there were moderate negative correlations for AF3 and P8 Theta. As shown in Table 13 (the number of errors in Task2 vs. the biological indexes during Rest2), there were moderate negative correlations for pNN90, Theta and Alpha of the frontal cortex (AF3, F4, and AF4) and Alpha and Low Beta of the right parietal lobe (P8). As shown in Table 14 (the number of errors in Task2 vs. the biological indexes during Task2), there was a moderate negative correlation for AF3 Theta. 

These results suggest that the participants who had many errors in Task2 had less activation on the frontal cortex not only during but also before the task (Task1 and Rest2), and had less activation on the right parietal lobe before the task (Task1 and Rest2), while less relaxed, as observed from the negative correlation of pNN90 before the task (Rest2). Compared to the results of Task1 described in Section 4.3, HRV indexes have significant correlations only before the task (Rest2) and it is a negative correlation. The reason for the difference is unclear, since this study did not change the order of Task1 (word reading) or Task2 (color answering); however, the correlations suggest that a proper relaxed condition is important to reduce errors regardless of the task’s cognitive load. Table 14 describes that there was only one during-task biological index that had a significant correlation, which means there were not many differences between participants who made many errors in Task2 and the participants who did not. Since the 11 biological indexes show a significant correlation with the number of errors of Task1, as described in Table 9, this result suggests that the pre-task condition is much more important for Task2 (high-cognitive-load color answering task), while both the pre-task and the during-task conditions are important for Task1 (low-cognitive-load word reading task).

The above results show the possibility of estimating the state in which error is likely to occur in Task2 (high-cognitive-load color answering task) from the biological indexes as follows: the activation of the frontal cortex (AF3) using Theta and the right parietal lobe (P8) using Theta during Task1, pNN90 and the activation of the frontal cortex (AF3, F4, and AF4) using Theta and Alpha and the right parietal lobe (P8) using Alpha and Low Beta in Rest2, and the activation of the frontal cortex (AF3) using Theta during Task2.

### 4.5. Result 4: Correlation Analysis between the Difference in the Number of Errors between Task1 and Task2 (Task2-Task1) and the Biological Indexes during Rest1, Task1, Rest2, and Task2

To clarify the relationship between the error caused by the cognitive load and the biological indexes, we defined the difference in the number of errors (Table 7) as the strength of the Stroop effect (the degree of the effect of cognitive load) and performed a correlation analysis between the difference in the number of errors and the biological indexes during each of the experiment phases Rest1, Task1, Rest2 and Task2. As described in Section 3.7, the correlations with EEG indexes during Rest1 were not analyzed because they were used for the normalization of EEG data during other experiment phases. Only biological indexes from HRV during Rest2 showed significant correlations as shown in Table 15.

Similar to the results of the number of errors at Task2 and the biological indexes in Rest2 (Table 13), there were moderate negative correlations for HRV indexes (pNN70, pNN80, pNN100, and HF) and P8 Low Beta, and strong negative correlations for pNN90. On the other hand, there was no significant correlation between the difference in the number of errors and the biological indexes during Rest1, Task1, and Task2. These results suggest that the number of errors influenced by the difference in cognitive load increases when participants are less relaxed, as observed from the negative correlations of pNNx (x > = 80) and HF, and the concentration is decreased, as observed from the negative correlation of the right parietal lobe (P8) Low Beta during the interval of the tasks. Comparing with the results described in Section 4.4, it is suggested that being in a relaxed condition while keeping the activation of the right parietal lobe between the low-cognitive-load task and the high-cognitive-load task can help decrease the number of errors occurred by the difference in cognitive load.

## 5. Discussion

Our experimental results indicate that the difference between Task1 and Task2 is not only the number of correct answers for word or color, but also the degree of cognitive load. The results of correlation analysis between the difference in the number of errors and the biological indexes suggest that the participants tend to be affected by the difference in cognitive load to make more errors in the high-cognitive-load task when their biological indexes during the pre-error condition before the high-cognitive-load task (Rest2) indicate the following states: a less relaxed condition based on pNNx and HF and a decreased concentration based on the right parietal lobe.

Since the number of biological indexes during Task2 that have significant correlations with the number of errors of Task2 is smaller than the number of biological indexes during Task1 that have significant correlations with the number of errors of Task1, there is a possibility that keeping a proper condition before performing a task is needed to reduce errors in a task with high cognitive load. On the other hand, maintaining a proper condition before and during the task may effectively reduce errors in a task with low cognitive load.

Table 16 and Table 17 summarize the correlation analysis results for HRV and EEG indexes, respectively. Interestingly, we obtained the positive correlations for HRV indexes only with the number of errors of Task1, while we obtained negative correlations for HRV indexes with the number of errors of Task2 and the difference in the number of errors between Task1 and Task2 (Task2–Task1). The EEG indexes, on the other hand, show moderate negative correlations with the number of errors in Task1, the number of errors in Task2, and the difference in the number of errors between Task1 and Task2. There are significant correlations between the number of errors of Task2 and both the pre-error condition of Task2 (Task1 and Rest2) and the during-task condition itself (Task2), while only the condition between Task1 and Task2 (Rest2) has a significant correlation with the difference in the number of errors.

From the correlation analysis results of HRV indexes (Table 16), it suggests that highly relaxed pre-task and during-task conditions observed from pNNx and HF, as well as the decreased concentration during tasks based on the activation of the frontal cortex and the right parietal lobe, may increase the human error probability during Task1 (low-cognitive-load word reading task). On the other hand, there is a possibility that the less relaxed pre-task condition, based on pNN90, the decreased concentration before the task, based on the activation of the frontal cortex and right parietal lobe, and the decreased concentration during the task, based on the activation of the frontal cortex may increase the number of errors of Task2 (high-cognitive-load color answering task).

From the correlation analysis results of EEG indexes (Table 17), the frontal cortex and the right parietal lobe are likely to play important roles in reducing errors. The frontal cortex is involved with cognition or judgement, and the right parietal lobe is involved with sensory function [53,58]. For the function of the right hemisphere, a study has reported that patients with right temporal lobe lesions have difficulty in visual processing tasks, while the left hemisphere is regarded to be related to language processing [55,69]. Considering these functions, the correlation analysis results of EEG indexes related to the frontal cortex and the right parietal lobe show that the errors were occurred not during language processing phase, but during visual processing, cognition or judgement phases.

These results show that we were able to predict the probability of human error for both of low- and high-cognitive-load tasks from HRV and EEG indexes of certain brain regions related to the error occurrence process.

There are some limitations in our experiment method, which are discussed below for the sake of future studies: 

Regarding the experimental procedure, the participants were asked to answer verbally during the Stroop task. It is known that while a person is speaking, the respiration and HRV tend to fluctuate, which may affect HRV interpretation. It is necessary to reconsider the answering method. In addition, we did not counterbalance the order of Task1 and Task2 to keep the same condition of cognitive level for all participants. To confirm the effect of those tasks themselves, we will counterbalance tasks in future studies. Moreover, we obtained results from participants in their 20 s, which is demographically biased. Since HRV and EEG patterns can vary by different age groups, we will recruit participants from a wide range of age groups in our future study. Finally, the participants had to answer each question in the Stroop task within 1 s, which may already cause high cognitive load and stress. Thus, we did not provide any feedback when the participants answered incorrectly because showing error may cause additional stress to her/him. However, since giving feedback may be necessary in some situations, our future work will also take into account the effect of feedback and what kind of feedback helps reduce stress caused by human error.

**Figure 5:**
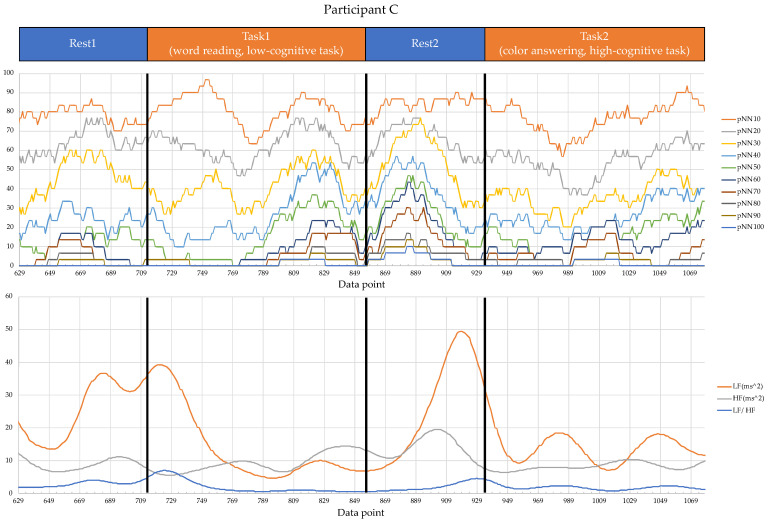
Raw waveforms used to calculate the HRV indexes of Participant C.

**Figure 6:**
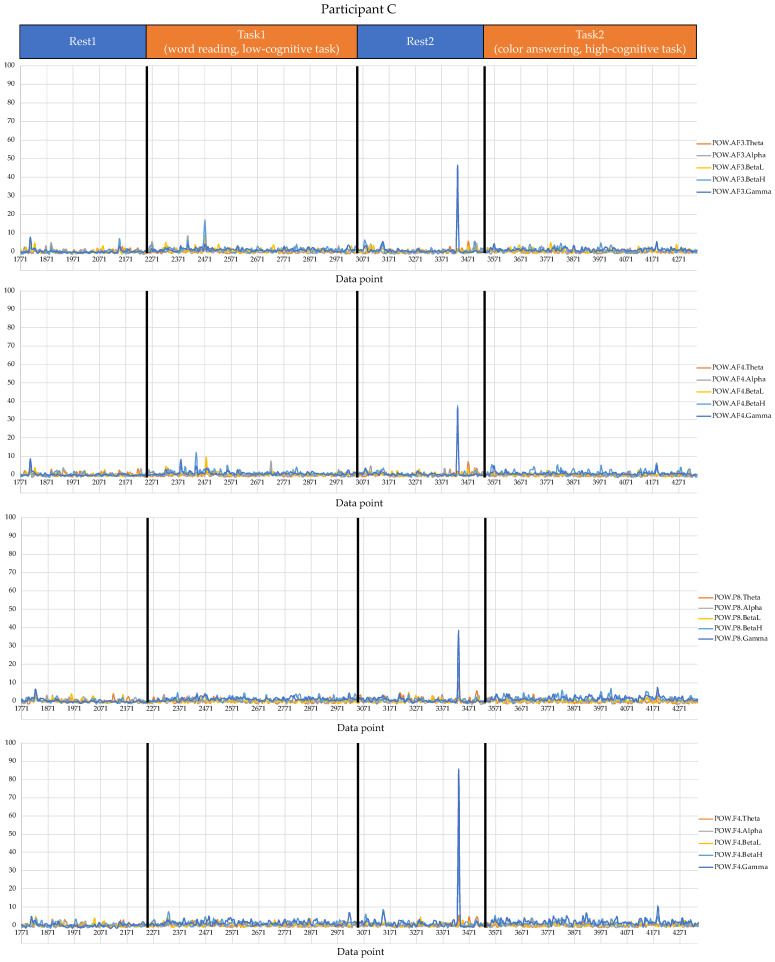
Raw waveforms used to calculate the EEG indexes of Participant C. (Note that the waveforms include only EEG channels with significant correlations (AF3, AF4, P8, and F4), as summarized in Table 17).

**Figure 7:**
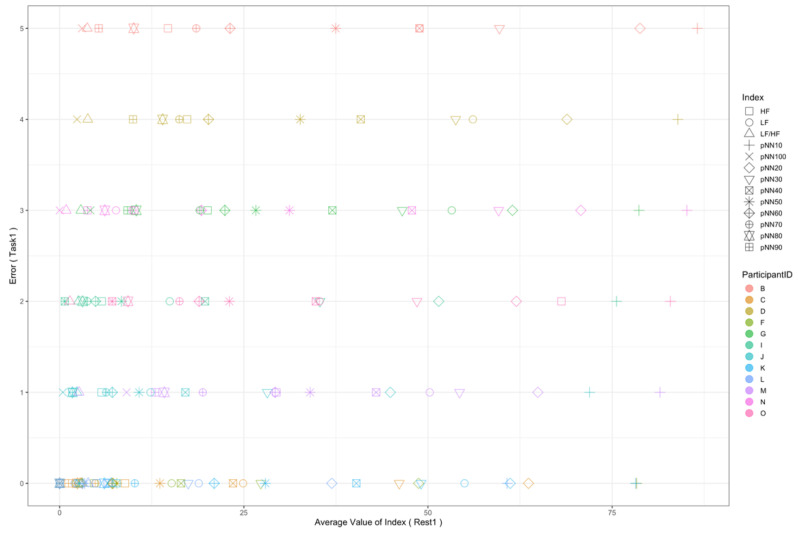
Scatterplot between the number of errors of Task1 vs. the HRV indexes during Rest1.

**Figure 8:**
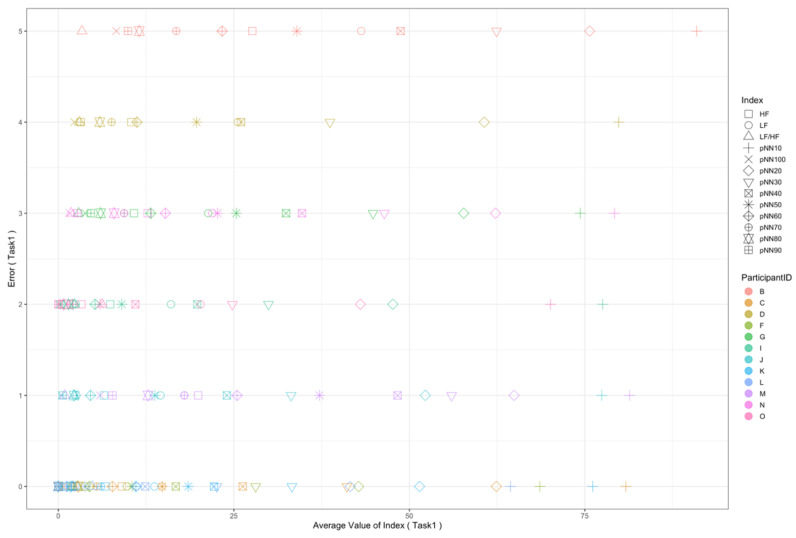
Scatterplot between the number of errors of Task1 vs. the HRV indexes during Task1.

**Figure 9:**
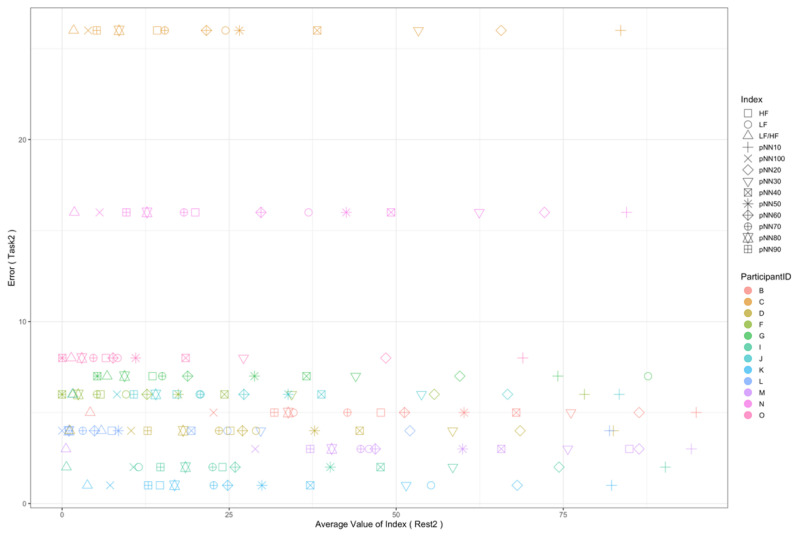
Scatterplot between the number of errors of Task2 vs. the HRV indexes during Rest2.

**Figure 10:**
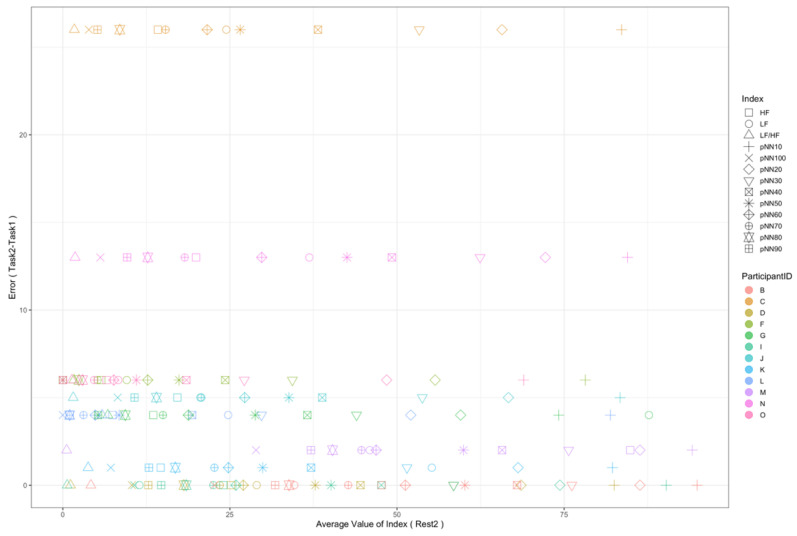
Scatterplot between the number of errors of Task2-Task1 vs. the HRV indexes during Rest2.

**Figure 11:**
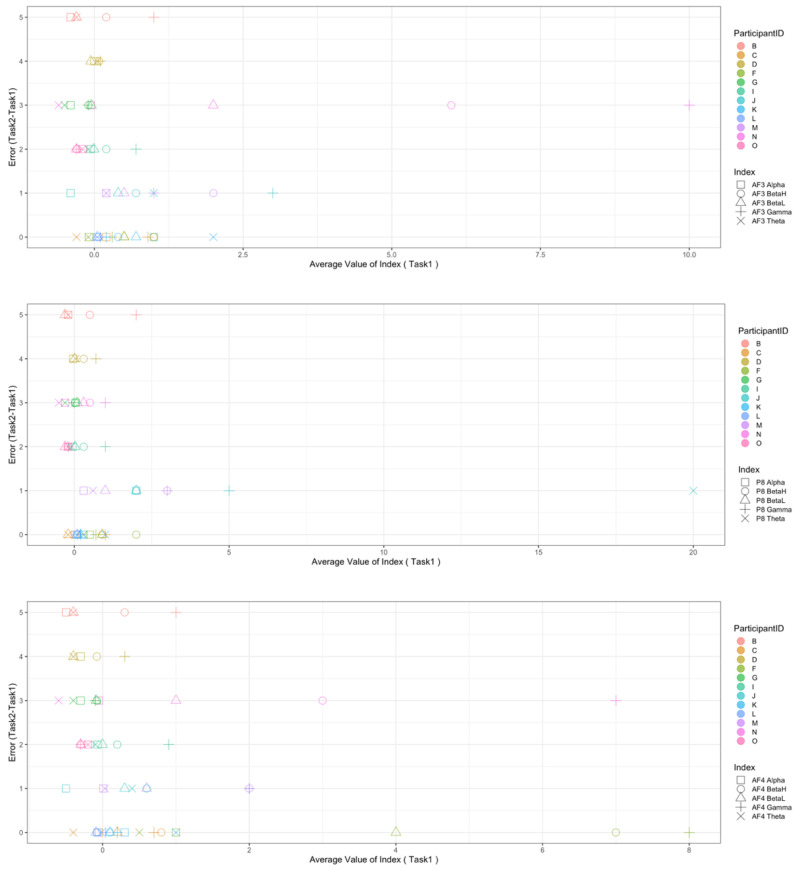
Scatterplot between the number of errors of Task1 vs. the EEG indexes during Task1. (Note that the scatterplots include only EEG channels with significant correlations (AF3, P8, and AF4) as summarized in Table 17).

**Figure 12:**
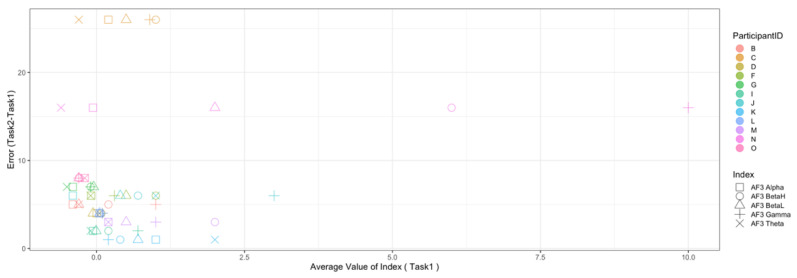

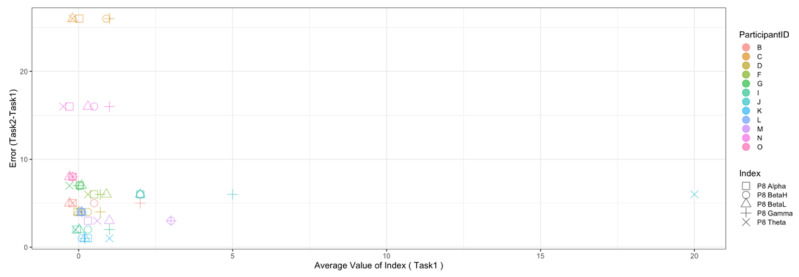
Scatterplot between the number of errors of Task2 vs. the EEG indexes during Task1. (Note that the scatterplots include only EEG channels with significant correlations (AF3 and P8), as summarized in Table 17).

**Figure 13:**
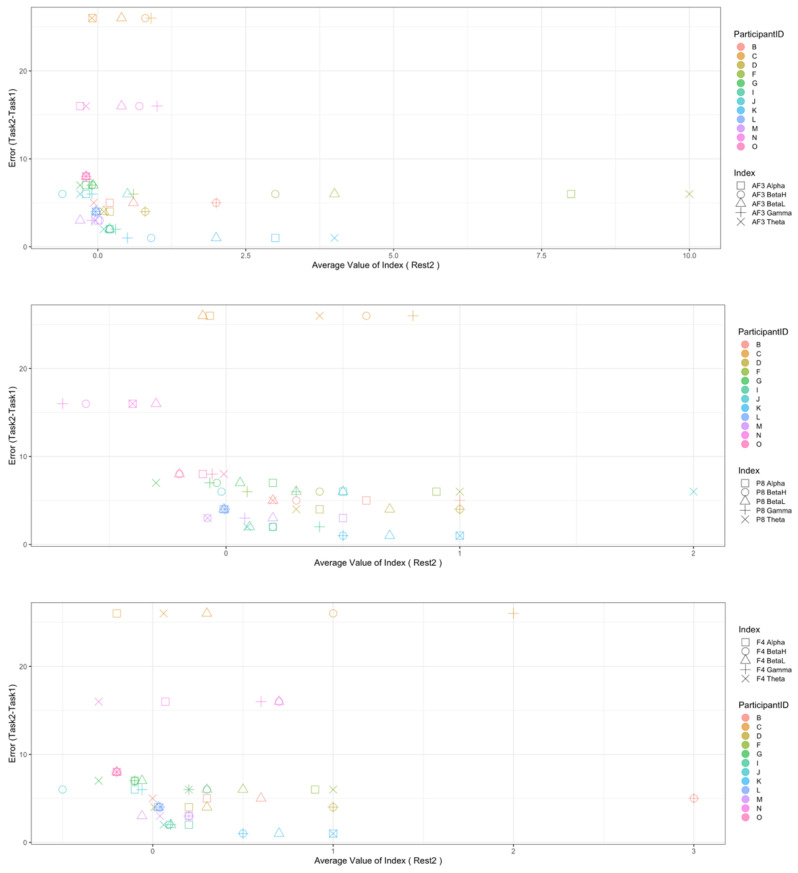

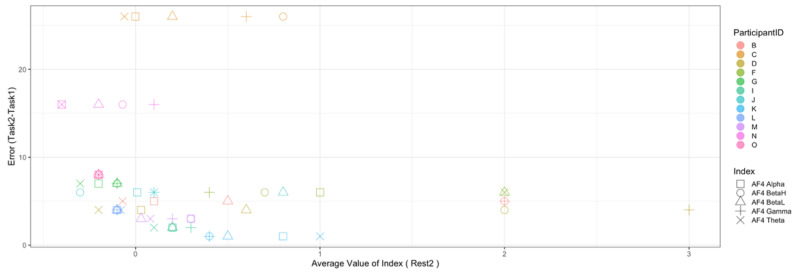
Scatterplot between the number of errors of Task2 vs. the EEG indexes during Rest2. (Note that the scatterplots include only EEG channels with significant correlations (AF3, P8, F4, and AF4), as summarized in Table 17).

**Figure 14:**
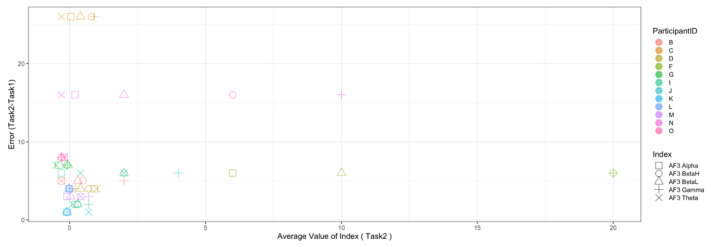
Scatterplot between the number of errors of Task2 vs. the EEG indexes during Task2. (Note that the scatterplots include only EEG channel with significant correlation (AF3), as summarized in Table 17).

**Figure 15:**
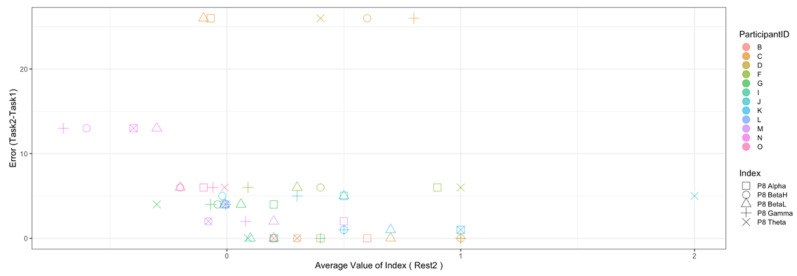
Scatterplot between the number of errors of Task2 vs. the EEG indexes during Rest2. (Note that the scatterplots include only the EEG channel with a significant correlation (P8), as summarized in Table 17).

Regarding the data analysis method, we did not analyze the correlation between the number of errors and the EEG indexes during Rest1 because the indexes were used for the normalization. We will further add a phase at the beginning of the experiment where EEG data are collected for normalization. In addition, since time-series data may contain interesting results related to changes in cognitive states, we will also include it in further data analysis. Though the time-series data analysis was not included in this manuscript, we provide the visualization of raw data, which may be used as a source to assess variance, signal clarity, etc. We provide an example of raw ECG and EEG waveforms of one participant (Participant C) as shown in Figure 5 and Figure 6, respectively. These raw data were used to calculate the HRV and EEG indexes used in this study. Furthermore, we provide scatterplots between the number of errors (horizontal axis) and the biological indexes (vertical axis) for each participant (indicated by each color) for the observation of intrapersonal variability among significant indexes. The scatterplots for HRV indexes are shown in Figure 7, Figure 8, Figure 9 and Figure 10. Those for EEG indexes are shown in Figure 11, Figure 12, Figure 13, Figure 14 and Figure 15. Note that the scatterplots only include EEG channels with significant correlations as summarized in Table 17. The detailed discussion and analysis of such intrapersonal variability will be included in further work.

## 6. Conclusions

To clarify the characteristics of biological indexes that can be useful to predict the state in which human error is likely to occur, we analyzed the relationships between the number of errors in the Stroop task and biological indexes before and during the task. We employed 14 HRV indexes and 70 EEG indexes in order to investigate multiple human error factors from biological indexes.

We found significant correlations between the number of errors in the Stroop task and the biological indexes, not only during but also before the task. In addition, there was a difference in the characteristics of the biological indexes between the low-cognitive-load task (word reading) and the high-cognitive-load task (color answering). For the low-cognitive-load task, HRV indexes obtained in pre-task and during-task conditions show significant positive correlations, while EEG indexes obtained in the during-task condition show significant negative correlations. On the other hand, for the high-cognitive-load task, pre-task HRV indexes show negative significant correlations, while pre-task and during-task EEG indexes show significant negative correlations. 

These results indicate the possibility that a relaxed internal state in pre-task or during-task conditions with decreased concentration may increase human error probability in low-cognitive-load tasks, whereas a less relaxed internal state in the pre-task condition with decreased concentration affects human error probability in high-cognitive-load tasks. Due to the limited circumstance of the experiment, we could collect and analyze the data from only 12 participants, which is a small number of statistical analysis in general. Despite of the small number of participants, we believe that the results at least give some clues on the order of importance among the biological indexes we employed, which will be worthwhile for the detailed study in the future. Our future work will continue to increase the number of participants as well as analyze the data in more detail to verify our results and establish a reliable method for predicting human error risk.

## Figures and Tables

**Table 1:** Previous studies using biological information and the targeted estimation of human states.

Biological Index	Human Psychological State Estimation	Human Error Probability Estimation	Human Error Occurrence Detection
HRV	Stress level [26] Cognitive capacity [29]	Mental calculation [25]	-
EEG	Workload [27] Cognitive flexibility [30] Teamwork [31]	Numerical typing [24] Finger-tapping task [37]	Modified flanker task [32] Eriksen flanker task [33] Error awareness dot task [34] Stroop task [35]
Multimodal indexes	Fatigue [20] Cognitive load [21] Difficulty of mental workload task [28]	Smartphone operation [39]	Stroop task [38]

**Table 2:** HRV indexes used in this study and their related psychological states.

HRV Index	Related Psychological State
RRI	PNS (vagal) stimulation and SNS stimulation [45]
pNNx	PNS activity [46,48]
LF	PNS activity, SNS activity, blood pressure, and baroreflex activity [47]
HF	PNS activity and respiratory cycle [47]
LF/HF	Sympathetic dominance [47]

**Table 4:** Correlation coefficients and related correlation strength [67].

Correlation Coefficient	Correlation Strength
|r| = 1.0	Perfect
0.7 < = |r| < 1.0	Strong
0.4 < = |r| < 0.7	Moderate
0.0< |r| < 0.4	Weak
|r| = 0.0	None

**Table 5:** Number of errors for each participant in Task1.

Participant ID	B	C	D	F	G	I	J	K	L	M	N	O	Avg.
Number of errors (Task1)	5	0	4	0	3	2	1	0	0	1	3	2	1.8

**Table 6:** Number of errors for each participant in Task2.

Participant ID	B	C	D	F	G	I	J	K	L	M	N	O	Avg.
Number of errors (Task2)	5	26	4	6	7	2	6	1	4	3	16	8	7.3

**Table 7:** Difference in the number of errors between Task1 and Task2.

Participant ID	B	C	D	F	G	I	J	K	L	M	N	O	Avg.
Number of errors (Task2–Task1)	0	26	0	6	4	0	5	1	4	2	13	6	5.6

**Table 8:** Correlation coefficients between the number of errors in Task1 and the mean values of biological indexes during Rest1 (the pre-error condition) (* correlation is significant at the 0.05 level; ** correlation is significant at the 0.01 level).

Biological Index	Correlation Coefficient	*p*-Value (2-Tailed)
pNN10	0.737 **	0.006
pNN20	0.673 *	0.017
pNN30	0.655 *	0.021
pNN40	0.669 *	0.017
pNN50	0.637 *	0.026
pNN70	0.580 *	0.048
pNN80	0.688 *	0.013
pNN90	0.627 *	0.029
HF	0.583 *	0.046

**Table 9:** Correlation coefficients between the number of errors in Task1 and the mean values of biological indexes during Task1 (during-error condition) (* correlation is significant at the 0.05 level; ** correlation is significant at the 0.01 level).

Biological Index	Correlation Coefficient	*p*-Value (2-Tailed)
pNN80	0.592 *	0.043
pNN90	0.595 *	0.041
pNN100	0.616 *	0.033
LF	0.941 **	0.000
HF	0.630 *	0.028
AF3 Alpha	−0.605 *	0.037
AF3 Low Beta	−0.608 *	0.036
P8 Theta	−0.587 *	0.045
P8 Alpha	−0.691 *	0.013
AF4 Theta	−0.698 *	0.012
AF4 Alpha	−0.644 *	0.024

**Table 10:** Correlation coefficients between the number of errors in Task1 and the mean values of biological indexes during the first half of Task1 (* correlation is significant at the 0.05 level).

Biological Index	Correlation Coefficient	*p*-Value (2-Tailed)
pNN80	0.635 *	0.027
LF	0.601 *	0.039
HF	0.576 *	0.050

**Table 11:** Correlation coefficients between the number of errors in Task1 and the mean values of biological indexes during the latter half of Task1 (* correlation is significant at the 0.05 level; ** correlation is significant at the 0.01 level).

Biological Index	Correlation Coefficient	*p*-Value (2-Tailed)
RRI	0.626 *	0.029
pNN10	0.601 *	0.039
LF	0.837 **	0.001

**Table 12:** Correlation coefficients between the number of errors in Task2 and the mean values of biological indexes during Task1 (pre-error condition) (* correlation is significant at the 0.05 level).

Biological Index	Correlation Coefficient	*p*-Value (2-Tailed)
AF3 Theta	−0.677 *	0.016
P8 Theta	−0.589 *	0.044

**Table 13:** Correlation coefficients between the number of errors in Task2 and the mean values of biological indexes during Rest2 (pre-error condition) (* correlation is significant at the 0.05 level).

Biological Index	Correlation Coefficient	*p*-Value (2-Tailed)
pNN90	−0.614 *	0.034
AF3 Theta	−0.642 *	0.024
AF3 Alpha	−0.649 *	0.022
P8 Alpha	−0.596 *	0.041
P8 Low Beta	−0.621 *	0.031
F4 Alpha	−0.691 *	0.013
AF4 Alpha	−0.684 *	0.014

**Table 14:** Correlation coefficients between the number of errors in Task2 and the mean values of biological indexes during Task2 (during-error condition) (* correlation is significant at the 0.05 level).

Biological Index	Correlation Coefficient	*p*-Value (2-Tailed)
AF3 Theta	−0.589 *	0.044

**Table 15:** Correlation coefficients between the difference in number of errors between the tasks and the mean values of biological indexes during Rest2 (* correlation is significant at the 0.05 level; ** correlation is significant at the 0.01 level).

Biological Index	Correlation Coefficient	*p*-Value (2-Tailed)
pNN70	−0.643 *	0.024
pNN80	−0.696 *	0.012
pNN90	−0.724 **	0.008
pNN100	−0.694 *	0.012
HF	−0.601 *	0.039
P8 Low Beta	−0.608 *	0.036

**Table 16:** Correlation analysis results of HRV indexes (“M+”: moderate positive correlation; “S+”: strong positive correlation; “M-”: moderate negative correlation; “S-”: strong negative correlation).

Number of Errors	Rest1 (Pre-Condition of Task1 and Task2)	Task1 (During-Task Condition of Task1 and the Pre-Condition of Task2)	Rest2 (Pre-Condition of Task2)	Task2 (During-Task Condition of Task2)
Task1 (low-cognitive-load word reading task)	pNN10 (S+)	pNN80 (M+)		
	pNN20 (M+)	pNN90 (M+)		
	pNN30 (M+)	pNN100 (M+)		
	pNN40 (M+)	LF (S+)		
	pNN50 (M+)	HF (M+)		
	pNN70 (M+)			
	pNN80 (M+)			
	pNN90 (M+)			
	HF (M+)			
Task2 (high-cognitive-load color answering task)	No correlation	No correlation	pNN90 (M-)	No correlation
Task2–Task1 (strength of the Stroop effect)	No correlation	No correlation	pNN70 (M-)	No correlation
			pNN80 (M-)	
			pNN90 (S-)	
			pNN100 (M-)	
			HF (M-)	

**Table 17:** Correlation analysis results of EEG indexes (“M-”: moderate negative correlation).

Number of Errors	Rest1 (Pre-Condition of Task1 and Task2)	Task1 (During-Task Condition of Task1 and the Pre-Condition of Task2)	Rest2 (Pre-Condition of Task2)	Task2 (During-Task Condition of Task2)
Task1 (low-cognitive-load word reading task)		AF3 Alpha (M-)		
		AF3 Low Beta (M-)		
		P8 Theta (M-)		
		P8 Alpha (M-)		
		AF4 Theta (M-)		
		AF4 Alpha (M-)		
Task2 (color answering, high cognitive task) Task2 (high-cognitive-load color answering task)		AF3 Theta (M-)	AF3 Theta (M-)	AF3 Theta (M-)
		P8 Theta (M-)	AF3 Alpha (M-)	
			P8 Alpha (M-)	
			P8 Low Beta (M-)	
			F4 Alpha (M-)	
			AF4 Alpha (M-)	
Task2–Task1 (strength of the Stroop effect)		No correlation	P8 Low Beta (M-)	No correlation
				AF3 Theta (M-)

## Data Availability

The data presented in this study are available from the corresponding author upon request. The data are not publicly available due to ethical reasons.
